# Case of Uterine Hemorrhage

**Published:** 1874-08

**Authors:** L. J. Willien

**Affiliations:** Terre Haute, Indiana


ARTICLE V.—*Uterine Hemorrhage, appearing after five and one-half Months' Gestation, caused by Detachment of the Placenta—Premature Delivery, favorable to Mother and Child.* By L. J. WILLIEN, M.D., Terre Haute, Indiana.

Knowing the anxiety and fear all practitioners entertain, when consulted or called to the bedside of women affected with hemorrhage, I will here give the history of one, rather of an interesting character.

Was summoned to see A., aged 16 years, medium size, nervoso-lymphatic temperament, delicately constituted; blonde, fair complexion. Has complained more or less of poor health during the last year. Menstruated when fourteen years of age; always regular, but accompanied every time with severe pains in back, and bowels, and headache, which soon ceased after the menses had appeared.

On the 18th day of May I made my first visit, and obtained the following information:

Menstruated the last time about the fifth of November, 1873; supposed to have become pregnant on or about the 15th of the same month; had enjoyed good health, no morning sickness, had good appetite, and bowels moved regularly. Never complained of pain until the latter part of April, when quickening increased, bearing-down sensation, pains in lumbar region, with more or less sensitiveness towards fundus of the womb, rather exaggerated on pressure; the discharges from the vagina, a muco-sanguinolent fluid, became daily more abundant, until they were followed by pure blood, leaving no doubt in our mind of threatening hemorrhage. Fœtal pulsations were distinctly heard over the pubis and to the right. External palpation revealed no abnormal condition, either in form or position of the uterus.

Per vaginal exploration: Vulva and vagina normal; perineal and constrictor muscles relaxed, the canal containing coagulated blood; cervix elongated, and os tincæ barely permitting the passage of the finger, yet from it exuded a small quantity of blood. By careful digital exploration we discovered no signs of tumor nor spongy substance on the inner surface of the os. Not being enabled to reach farther at the time, we desisted from using force or subjecting the patient to more fatigue. The question as to the diagnosis was, Had we a case of placenta previa, *i. e.*, a partial one, or a detachment of the placenta's outer edge?

A few days of close attention and patience soon revealed to us that it was the latter.

In the meantime we ordered our patient to absolute rest, cold, acid draughts, bowels moved with siedlitz powders, laudanized enema per rectum, and 30 drops of fl. extr. of ergot every three or four hours, until hemorrhage ceased. On the 19th and 20th, patient is somewhat better of pains, but flooding continues and is becoming more abundant. The same evening at 4 P. M. the patient was taken with a chill, followed by severe uterine contractions. Ordered sulph. of quiniæ, gr. ij; ergotine, gr. iij; ext. conii, gr. j; M. ft. pil. No. 1. Repeat every three hours; having suspended ergot several days previously.

After taking the pill the patient felt much relieved, and hemorrhage apparently ceased. On 21st and 22d, rested very comfortably; only complained of a little nausea toward bedtime.

May 23, at 6 P. M., hemorrhage becomes more abundant, uterine contractions continual, with unrelenting pains in the back, general nervous jactation, hands and feet cold, pulse 100, soft and regular, facies turgescent; considerable blood in vagina, cervix effaced, os tincæ flattened and edges yet resistant; head presses down.

Ordered enema with 20 m. tr. opii, per rectum; R. Inter. gum opii, gr. $\frac{3}{4}$; pulv. pot. chlor., gr. vj; pulv. pot. bromidi, gr. xv. To be repeated in sweetened water every three hours until relieved.

Soon after, patient fell asleep and rested until morning. Fearing perhaps some intermittent, rheumatismal type of uterine fibres, we ordered 8 grs. of quinine to the above powder, which the patient took on the morning of the 24th. Called to visit her again at 11 A. M. Wasted very little during the night. Vomited early in the morning, but afterwards retained her victuals. Uterus hard and painful on pressure. Pulse soft and regular; 80 per minute. Bowels constipated. Ordered seidlitz powder every two hours, and absolute rest.

At 3 o'clock P. M. was summoned in haste to her bedside, with information that she was worse. Fearing any serious complications, and unwilling to take upon ourselves all responsibility, we kindly invited Dr. J. D. Mitchell to visit the patient with us. At our arrival we found uterine contraction fully established, strong, regular and expulsive. On external palpation felt strong motions of the child. The vagina was moist, more or less blood flowing therefrom; cervix uteri completely effaced; os tincæ dilated to the size of a silver quarter; head of child pressing into the upper strait; membranes protruding slowly. No signs whatever of placenta juxta ori being discovered.

Six P. M. Os dilated, membranes protrude, contractions strongly expulsive, membranes give, and are ruptured at half-past seven. The head passes through the vulva at eight o'clock. The child breathed immediately; of the female sex; very small, of about two and a half pounds' weight; body well developed; finger and toe-nails nearly grown. The liquor amnii was abundant, and of a dark-brown color. The placenta was expelled a few seconds after the birth of the child, and discovered the following points of interest:

1st. Funis twelve inches long; umbilical veins varicosed; placentary tissue very consistent, oval-shaped, showing near the margin to the extent of three inches in length and about an inch and a half in width, a dark, soft mass of previously detached placental tissue; the utero-placentar vessels gradually breaking, and thereby causing the hemorrhage. Not the least sign of flooding appeared after delivery, both mother and child doing well.

This premature delivery we considered a happy event for the mother and child, when we know how alarming and dangerous uterine hemorrhages are, no matter from what cause, during gestation.

